# Research progress on Sertoli cell secretion during spermatogenesis

**DOI:** 10.3389/fendo.2024.1456410

**Published:** 2025-01-15

**Authors:** Yao Xiao, Jingyi Zhang, Yanxin Guan, Meijing Wang, Dehong Liu, Shengxi Xiong, Junjun Li, Xujun Yu

**Affiliations:** Sichuan Provincial Key Laboratory of Traditional Chinese Medicine Regulation of Metabolic Diseases, Hospital of Chengdu University of Traditional Chinese Medicine, Chengdu, Sichuan, China

**Keywords:** spermatogenesis, germ cells, Sertoli cells, lactic acid, AMH, cytokines

## Abstract

Sertoli cells (SCs), as the somatic cells in the testis of male mammals, play a crucial role in the close association with germ cells. The blood-testicular barrier (BTB), established by their tight junctions, provides immune protection to germ cells, leading to their characterization as "sentinel" cells. Moreover, the physiological process of testicular development and spermatogenesis in male animals is intricately tied to the secretory activities of SCs. These cells secrete a diverse array of proteins and cytokines that interact with various targets, working in concert with mechanisms in the spermatogenesis pathway and contributing to each stage, from spermatogonial cell division to the maturation of spermatozoa. Hence, the secretory products of SCs are pivotal in fostering germ cell development and directing the appropriate maturation of sperm. This study is dedicated to investigating the varied secretions of SCs, outlining their critical functions throughout distinct phases of spermatogenesis, thus elucidating the substantial influence of SC secretion on male fertility. Furthermore, it offers valuable perspectives on reproductive disorders stemming from irregular spermatogenesis in clinical contexts.

## 1 Introduction

Spermatogenesis is a dynamic development process under strict regulation by multifaceted genetic mechanisms, and germ cells need to undergo continuous coordinated morphological transformation of spermatogonia (mitosis), primary spermatogonia (meiosis) and secondary spermatogonia (postmeiotic stage) to produce mature sperm (1). Classification in Ad (spermatogonial stem cells), Ap (differentiated) and B type applies to primates, such as monkey and man. The Ap and Ad spermatogonia are considered to be the counterpart of the mouse undifferentiated spermatogonia, and type B that of the mouse differentiating spermatogonia.During the premeiotic phase, primordia germ cells adhere to the basement membrane, consisting of Ad, Ap and B type. While Ad types remain undifferentiated, Ap types typically differentiate into two B type spermatogonia (2). Type B spermatogonia undergo mitosis to generate primary spermatocytes (3). Subsequently, spermatocytes progress through the first meiotic division, resulting in the formation of two secondary spermatocytes. These secondary spermatocytes undergo successive meiotic stages, culminating in a second meiotic division, ultimately yielding two round haploid sperm cells devoid of further division activity. Through intricate spermatogenic processes, these cells further differentiate into spermatozoa of varying lengths (4). Disruption at any stage of this cascade impairs the formation of mature sperm.

Sertoli cells (SCs), as somatic cells within the testis, serve as the principal structural elements of the spermatogenic tubules. The distinctive junctional complexes formed by SCs create a supportive framework and microenvironment for germ cell growth, offering essential "nutrients" and support for testicular development and spermatogenesis (5). Their roles in directing spermatogenesis, conferring immune protection to germ cells, supplying nutrients, and managing waste underscore the pivotal contribution of SCs to the process of spermatogenesis (6). The effective execution of supporting cell functions is intricately linked to the generation of viable sperm. Consequently, it holds paramount importance to delineate the distinct roles of secreted substances across diverse stages of spermatogenesis in order to elucidate the pathological mechanisms underlying disorders in spermatogenesis.

## 2 Sertoli cell secretions acting on meiosis prophase

### 2.1 Stem cell factor

Stem cell factor (Scf) is expressed in SCs, endothelial cells, stromal cells and other testicular cells. Inhibition of spermatogonial differentiation occurs upon SCF knockdown in SCs, whereas overexpression of SCF leads to a substantial augmentation in spermatogenesis. These findings underscore the indispensable role of SCF secreted by SCs in the regulation and preservation of spermatogonial differentiation (7). In a rat model of testicular atrophy induced by 2,5-hexanediol (2,5-HD) treatment, the predominant form of SCF mRNA transitioned from the transmembrane form to the soluble form following 13 weeks of intervention. Subsequently, SCF mRNA exhibited enrichment concomitant with germ cell depletion after 22 weeks. Treatment with SCF (100-200 μg/kg BW) resulted in an increase in the number of germ cell queues, an elevation in the count of BRDU-positive cells, and an expansion of the positively labeled cell population. SCF plays an important role in maintaining the vitality of A2-A4 spermatogonocytes and is essential for the differentiation and proliferation of spermatogonocytes (8). Through the Kit/SCF-R signaling pathway, SCF activates phosphoinositide 3-kinase (PI3K) sites to modulate the proliferation and differentiation of spermatogonocytes (9). In in vitro co-culture experiments involving the isolation of SSCs and SCs from human fetal testes, it was observed that 100 ng/ml exogenous E2 stimulated the transcription of the SCF/c-kit signaling pathway with SCF as the central mediator. This led to the augmentation of 8-16 or more spermatogonial stem cell colonies within SCs, thereby modulating the proliferation of fetal spermatogonial stem cells and inhibiting apoptosis (10). Additionally, Feng et al. demonstrated that SCF/c-kit regulates spermatogonial proliferation through the PI3K/AKT/p70S6K/cyclinD3 pathway (11). In the rat model of varicocele (VC), a significant decrease in the number of cells within the spermatogenic tubules of VC rats compared to the control group was observed. Furthermore, an elevation in the concentration of SCF in the testis was noted, while the expression intensity of c-kit was lower in the VC group than in the control group, suggesting that the dysregulation of SCF/c-kit system may frequently be associated with VC-induced spermatogenesis (12).

The Bcl-2 family comprises proteins that regulate mitochondrial outer membrane permeability. Intervention of SCF in the spermatogenic tubule system resulted in varied alterations in the expression levels of pro-apoptotic members of the Bcl-2 family (such as Bax, Bad, Bak and Bok) and anti-apoptotic proteins (such as Bcl-2 proper, Bcl-xL, and Bcl-w). In the initial phase of spermatogenesis, SCF initially utilizes p53 to facilitate the re-expression of Bax and Bad, thereby triggering germ cell apoptosis. Subsequently, SCF modulates the dynamic expression of the Bcl-2 family by upregulating anti-apoptotic proteins (e.g., Bcl-xL, Bcl-w) and downregulating pro-apoptotic proteins (e.g., Bax) to promote the survival and viability of germ cells throughout spermatogenesis (13). SCF intervention in spermatogenic tubule cultures during phase XII resulted in a decrease in germ cell apoptosis. SCF utilizes follicle-stimulating hormone (FSH) stimulation to activate protective mechanisms in spermatogonia, spermatocytes, and sperm cells (14). In the culture of adult testicular fragments, a notable distinction was observed: the proportion of eighth-generation cells (primary spermatocytes) markedly increased after 3 weeks in the culture medium supplemented with FSH, while the culture medium supplemented with SCF did not consistently exhibit the presence of primary spermatocytes. While SCF can stimulate spermatogonial proliferation in male salamanders, it is unable to initiate spermatogonial progression to meiosis; instead, it directly induces apoptosis in this cell population (15).

### 2.2 Exosomes

Exosomes released by SCs play a crucial role in spermatogenesis. Polyvesicles, also known as multivesicular bodies (MVB), release exosomes upon fusion with the plasma membrane. As a communication vector, Sertoli cells utilize this exosome-MVB pathway to transport a diverse array of proteins, nucleic acids, and lipids to both round spermatogonocytes and primary spermatogonocytes, thereby maintaining the normal homeostasis essential for spermatogenesis (16). Studies have shown that the exosome-associated ribonuclease Dis3 plays a crucial role in stabilizing RNA levels and gene expression coordination in spermatogonocytes. It achieves this by maintaining a delicate balance between RNA synthesis and degradation, thereby ensuring the integrity of spermatogenic lineages and supporting the normal development of spermatogonocytes (17). Exosomes released by Sertoli cells regulate the differentiation of spermatogonial stem cell through a paracrine mechanism involving neighboring spermatogonial stem cells, as well as an autocrine effect that influence the GDNF expression signal in the supporting cells themselves. However, the specific mechanism by which SCs-EXO regulate spermatogonial stem cell differentiation remain poorly understood (18). Sertoli cells manage the secretion of extracellular vesicles (EVs) through the palmitoylation of autovacuolar membrane protein 1 (VMP1), thereby regulating the niche and self-renewal of spermatogonial stem cells (19). Concurrently, other studies have uncovered that exosomes secreted by SCs can deliver miR-30a-5p to spermatogonial stem cells, where it regulates the MAPK pathway by targeting the zinc finger E-box binding homeobox 2 (ZEB2), thereby promoting the differentiation and proliferation of spermatogonial stem cells (4) It has been demonstrated that a hypoxia environment induces the secretion of hypoxy-sensitive miR-210-3P by Sertoli cells, which may serve as a significant biomarker for assessing the extent of Sertoli cell damage in patients with varicocele (20). Acting as a bridge between Sertoli cells and spermatogonial stem cells, exosomes play an important role in the process of spermatogenesis. However, current research into exosomes secreted by Sertoli cells is not yet comprehensive and requires further exploration.

### 2.3 Cytokines

Glial-cell-line-derived neurotrophic factor (GDNF) secreted by SCs serves as the genuine self-renewal factor for spermatogonial stem cells (21). In a mouse model with Cdc42-deficient SCs, the MAPK1/3 scaffold proteins pMAPK1/3 (phosphorylated MAPK1/3) and PEA15A translocate from the SC nucleus to the cytoplasm, impeding the nuclear localization of MAPK1/3 within SCs. Consequently, the activation of the inhibitory signal of the MAP2K1 pathway ensues, leading to reduced expression of the SC transcription factors DMRT1 and SOX9. This downregulation subsequently impacts the expression of GDNF, culminating in spermatogenic abnormalities in CDC42-deficient mice (22). In a study by Ibtisham et al. (23), tissue fragment cultures were performed on the testes of newborn piglets for a duration of 8 weeks. The results revealed that the intervention of GDNF in the in vitro spermatogenesis (IVS) model exhibited superior maintenance of spermatogenic tubule integrity, as well as enhanced vitality and quantity of germ cells, when compared to the control group and other growth factors. Supplementing the culture with GDNF led to an increased proportion of spermatogonia within the spermatogenic tubules. GDNF plays a crucial role in preserving early testicular germ cells in pigs. In a SC-spermatogonial stem cell co-culture experiment involving 3 to 6-month-old buffalo testes, a growth factor combination comprising GDNF, FGF-2, and EGF was employed to treat the buffalo testis cell co-culture system. Following the establishment of SSC colonies, there was a notable increase in both the area and enrichment of SSCs in the treatment group in comparison to the control group. Moreover, the expression levels of certain miRNAs associated with self-renewal and apoptosis (miR-20b, miR-21, miR-106a) in SSCs were significantly elevated in the treatment group as opposed to the control group (24). The growth factors secreted by SCs intricately modulate the expression of SSC self-renewal and apoptosis through the upregulation of associated miRNAs. This regulation helps sustain the stem cell characteristics of SSCs, thereby fostering a conducive environment essential for spermatogenesis. Inhibition of the signal transduction pathway of GDNF results in significant damage to 90% of SSCs. However, restoration of the normal GDNF signal prompts the initiation of regeneration by spermatogonia (25). Thus, it can be inferred that GDNF plays a crucial role in the self-renewal, growth, and development of SSCs.

## 3 Sertoli cell secretions acting on meiosis

### 3.1 Transferrin

According to reports, the peak expression stage of Transferrin (Trf) mRNA in the testes of adult rats occurs during the second half of the spermatogenic epithelial cycle, specifically in stages VIII-XIV. Following a 7-day treatment with methoxyacetic acid (MAA), there was a slight yet noteworthy elevation in Trf mRNA levels coinciding with the reduction of spermatocytes in the pachytene stage of the spermatogenic epithelium (26). The expression of Trf was found to be negatively impacted by spermatocytes in the pachytene stage. In male rats exposed to bisphenol A (BPA) and di (2-ethylhexyl) phthalate (DEHP) during the embryonic period, a notable reduction in the expression levels of Trf was observed in supporting cells. This reduction was associated with pathological changes in spermatogenic epithelial cells, characterized by extensive atrophy and disintegration of spermatogenic tubules (27). C. Lécureuil et al. (28) generated transgenic mice expressing human transferrin (hTrf) and observed that the Trf mRNA levels in these mice were over two times higher compared to wild-type mice. Furthermore, the secretion of transferrin in primary SCs from wild-type mice increased in a dose-dependent manner upon stimulation with 10-500 μg/ml hTrf. In the context of Trf overexpression, the testes and epididymides of hTrf transgenic mice exhibited consistent weights between 3 and 16 months of age. However, by 16 months of age, the testes and epididymides of hTrf transgenic mice were smaller compared to those of wild-type mice, with a notable reduction in testicular sperm reserves. Additionally, the levels of estradiol-17beta (E2-17β) were elevated to twice that of wild-type mice in the hTrf transgenic mice. Nevertheless, spermatogenesis and fertility remained unaffected. The excessive expression of Trf induced by hTrf in mice resulted in heightened secretion of supporting cell fluid, ultimately causing testicular dysfunction in mice during late development.

In VC rats, the immunolabeling of Trf in the spermatogenic epithelium was notably diminished compared to normal rats. Particularly during the androgen-dependent stage of the spermatogenic epithelial cycle (VII-VIII), the expression of Trf exhibited a more pronounced downregulation. Furthermore, the extent of spermatogenic impairment in VC rats was positively associated with the rate of inhibition in Trf expression (29). In a clinical study involving enzyme immunoassays of 130 semen samples, it was observed that the levels of Trf and soluble Trf receptor (S-Trf-R) in the semen of patients with oligospermia, asthenospermia, azoospermia, and post-vasectomy were markedly lower compared to those in the normal sperm group (30).

### 3.2 ABP

Androgen-binding protein (ABP) expression was dose-dependently induced by estradiol (31), with a significant increase observed under 0.1 nM E2 intervention and a decrease noted under 10 nM E2 intervention. D. M. Selva et al. (32) generated mice overexpressing ABP by developing mice harboring the rat ABP (rABP) /sex hormone-binding globulin (SHBG) clone gene. In comparison to the control group, homozygous and heterozygous mice exhibited a decrease in round and elongated sperm cells (haploid), along with a notable increase in primary spermatocytes (tetraploid) at the spermatogonial, leptotene, zygotene, and pachytene stages. Furthermore, there was a significant elevation in the number of apoptotic cells. The overexpression of ABP led to the accumulation of a large quantity of spermatocytes and sperm cells in the pachytene stage within the spermatogenic tubules, resulting in cellular degeneration and arrest at the first meiosis stage. In a study by Yu et al. (33), it was demonstrated that intragastric administration of 100/200 mg/kg carbendazim to male rats for 60 days induced excessive secretion of ABP through competitive binding with ABP. This excessive ABP production caused a significant increase in apoptosis of spermatocytes in the pachytene stage, resulting in reproductive system damage and a marked decline in sperm quality in rats.

The expression level of ABP effectively mirrors the vitality and functionality of supporting cells (34). Analysis of testicular tissue biopsies from patients with maturation block and SC-only syndrome revealed a significant downregulation in ABP mRNA expression, leading to impaired development of SC-only cells (35).

### 3.3 Statin

As a glycoprotein hormone originating from the gonads, inhibin acts on pituitary gonadotropins and suppresses the secretion of follicle-stimulating hormone (FSH), thereby playing a crucial role in preserving the normal gonadal function. The concentration of inhibin B (INHB) is intricately linked to the quantity and function of pachytene spermatocytes and round spermatocytes, although it does not directly correlate with the presence of mature spermatozoa (36). An endocrine investigation involving 100 adolescent boys revealed that during the initiation of spermatogenesis in middle adolescence (G3-G4), a rise in INHB levels corresponded with the culmination of spermatogenesis. Additionally, a negative association between serum INHB and follicle-stimulating hormone (FSH) gradually emerged, indicating the normal progression of spermatogenesis (37). Similarly, an examination of INHB levels in 89 patients with azoospermia stemming from various testicular pathologies revealed a notable discrepancy in INHB levels among patients with spermatogonial/spermatocyte stasis (48.9 ± 16.7) when contrasted with the control group (148.5 ± 46.8). This observation suggests a correlation between INHB levels and abnormalities in sperm meiosis (38). In the testis of prepubertal boys, SCs exhibit robust positive signals for both the inhibin alpha (INHα) subunit and the inhibin beta B (INHβB) subunit. However, in mature male testes, SCs solely display the signal for the INHα subunit, while the signal for the INHβB subunit is evident in germ cells (specifically pachytene spermatocytes to round spermatocytes) (39). The post-pubertal secretion of INHB is likely regulated by both SCs and germ cells. The findings from the experiments conducted by C. Marchetti et al. (40) reaffirmed the notion that adult INHB may be a collaborative output of both supporting cells and germ cells. This observation also elucidated the molecular underpinnings behind the close association between adult male spermatogenesis and the level of INHB secretion.

During the assessment and monitoring of hormone levels in 100 patients with primary infertility, it was observed that sperm count exhibited a robust positive correlation with INHB concentration and underwent negative feedback regulation with FSH. Among 59 patients who achieved successful pregnancies, the INHB concentration was notably lower than normal; however, the natural pregnancy rate reached 81%. There were instances where both INHB and FSH levels fell within the normal range. While INHB concentration can serve as a marker for spermatogenesis, its clinical relevance remains constrained, and it should not be solely relied upon as an indicator of individual patient fertility (41). Following exposure of male mouse offspring to 4.8 and 43.2 mg/kg bw PM2.5, a significant shedding of spermatogenic cells from the spermatogenic tubules was observed, along with evident vacuolation of SCs. Methylation of INHB can trigger the activation of the p21/Cleaved Caspase-3 apoptotic pathway, leading to the extension of SC stasis and dysspermatogenesis (42). In the rat model of left VC, the sperm count in the left testicle of the experimental group was reduced, accompanied by a downregulation in INHB mRNA expression. Furthermore, an upregulation in Fas/FasL and caspase-3 mRNA expression was notably observed, particularly concentrated in spermatogenic cells. This molecular alteration correlated with an elevated apoptosis index of spermatogenic cells (43). VC formation triggers the activation of the Fas/FasL pathway, initiating the apoptosis cascade in SCs and germ cells, which in turn reduces the secretion of INHB and culminates in infertility. Through intricate pathway interactions, INHB influences the aberrant development of sperm meiosis, consequently contributing to spermatogenic failure.

### 3.4 Activators

During the initial phases of spermatogenesis, diminished activin A signaling diminishes the repression of the KIT-KITL pathway, impacting the proliferation of supporting cells and influencing spermatogonial differentiation (44). In in vivo models, the expression of meiotic germ cell markers (Sycp3, Ccnd3, Dazl) in the testes of homozygous transgenic mice with reduced activin bioactivity exhibited varying degrees of elevation compared to wild-type mice. In homozygous transgenic mice 7 days postnatal, there was an increase in the proportion of differentiated spermatogonia, alongside a decrease in the proportion of undifferentiated spermatogonia and somatic cells (including SCs). By day 14 postnatal, the numbers of spermatocytes, total germ cells, and total SCs in the testes of homozygous mice exhibited varying degrees of decline. Specifically, the counts of type A and type B spermatogonia decreased to 28.6% and 15.7% of those in wild-type mice, respectively. The sustained elevation of Activin-A (ACV-A) expression in the testes of adult Inha knockout (Inha KO) mice resulted in an augmentation of the proportion of SSC within tubule spermatogonia, alterations in spermatogonial cell clusters, enhanced proliferation of GFRA1+SSC enriched populations, and the establishment of a microenvironment conducive to SSC self-renewal (45). In in vitro models, the mRNA expression of Kit (a marker of germ cell maturation) exhibited a pronounced negative feedback response to activin doses of 50ng/ml and 100ng/ml in testicular Sertoli-germ cell co-cultures derived from 8-day-old mice. Additionally, the level of Activin-A in rat testes transiently peaked at 6 days postnatal and underwent a sharp decline by 15 days postnatal. The expression pattern of ACV-A corresponded to the phases of supporting cell proliferation and the cessation of the proliferation period. ACV-A potentially interacts with FSH to regulate supporting cells and influence sperm production (46). In In a dedicated in vitro culture of pachytene spermatocytes, dosages of 0.25 and 0.5 ng/ml of ACV-A demonstrated a dose-dependent elevation in the ratio of "condensed" mitochondria within primary spermatocytes. Moreover, the inclusion of ACV-A-specific antisera effectively nullified this rise in "condensed" mitochondria, thereby reinstating the ratio to pre-ACV-A intervention levels (47). ACV-A exerts a specific influence on germ cell meiosis. In experimental autoimmune orchitis (EAO) mice, the upregulation of TNF prompted a substantial release of ACV-A by SCs. During this period, ACV-A facilitated the phosphorylation and nuclear translocation of pSMAD2 in peritubular cells (PTCs) and fibroblasts, leading to the upregulation of fibronectin and collagen expression. This process resulted in elevated levels and deposition of fibronectin and collagen, contributing to atrophy of the lamina propria and spermatogenic tubule. The significant positive correlation between ACV-A and the severity of testicular inflammation highlights ACV-A as a potential target in testicular inflammatory conditions (48). IL-1 induces a dose-dependent increase in Activin A (ACV-A) secretion from immature SCs, with concomitant regulation by FSH inhibition, thereby modulating ACV-A secretion across the spermatogenic epithelial cycle (49). ACV-A is detectable throughout the spermatogenic epithelial cycle, with its specific expression commencing an ascent in the VIIcd phase and reaching peak secretion levels in the VIII phase. In contrast, INHB levels peak during the XI-I phase and exhibit slightly lower levels in the IV-VII phase. The peak expression phase of ACV-A aligns with the nadir of INHB secretion, declining concurrently during stages I-VI and XIV. The cyclic regulation of ACV-A and INHB throughout the spermatogenic epithelial cycle is orchestrated in part by endogenous IL-1α involvement (50).

### 3.5 Tumor necrosis factor

In rat testis, during the late VII to early VIII stages of the epithelial cycle, the primitive type A and type B spermatogonia undergo differentiation into the aligned spermatogonia and primary spermatocytes, transitioning towards the basal compartment within the seminiferous tubules, marking progression in the spermatogenic process. Research indicates that tumor necrosis factor alpha (TNF-α) exhibits specific localization solely within the epithelial cycle stages VI to early VIII. Following acute administration of 2mg/testis TNF-α within a physiologically relevant range (indicative of non-toxic effects), the expression of tight junction (TJ)-associated integral membrane proteins, including occludin and TJ-associated protein ZO-1, significantly declined by the third day. Conversely, the expression of other integral membrane proteins, such as JAM-A, remained unaffected, indicating normal cellular function.

The cellular layers, sperm cells, and spermatogenic epithelium exhibit reduced thickness, while the TJ of the blood-testis barrier (BTB) undergo severe damage. This damage results in significant enlargement of the intercellular spaces between SCs, leading to disrupted SC-SC adhesion and impaired SC-germ cell (GC) interactions. Nevertheless, the reductions in occludin, ZO-1, and N-cadherin levels were restored by the 8th day (51). These findings suggest that SCs release TNF-α into the BTB microenvironment during the epithelial cycle stages VI to early VIII, causing a rapid disruption in the BTB integrity by downregulating the expression of key proteins (occludin, ZO-1, N-cadherin). Assisting late VII-early VIII stage spermatocytes in transitioning from the basal layer to the proximal luminal layer is essential for advancing to the subsequent stages of sperm development. Following this migration completion, targeted protein damage recovery is crucial for the restoration and reconstruction of the intact BTB.

## 4 Sertoli cell secretions acting on meiosis anaphase

### 4.1 Lactic acid

**Figure 1:**
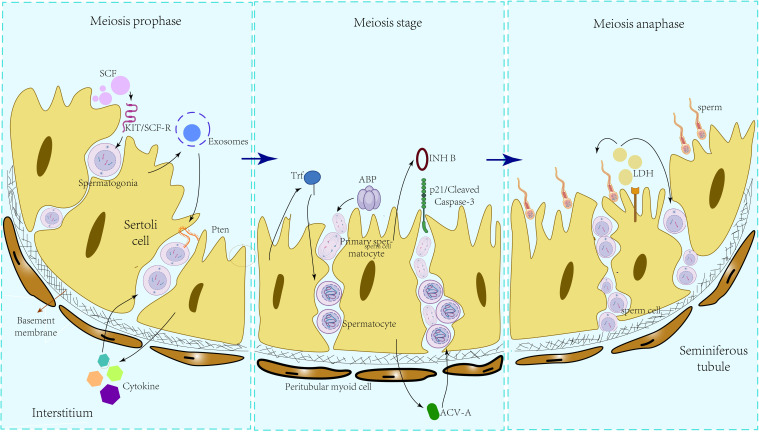
Influence of different SC secretions on various stages of spermatogenesis.

Lactate dehydrogenase (LDH) in the testes is secreted by SCs, with spermatocytes and mature sperm cells utilizing LDH as a fuel source for ATP production. Research (52) has demonstrated the enrichment of LDHA expression in SCs, and conditional knockout experiments targeting LDHA in spermatogenic cells and SCs revealed that the absence of LDHA in spermatogenic cells had minimal impact on spermatogenesis and fertility. In the LDHA knockout model group, there was a reduction of 34.12% and 46.0% in the proportion of grade A and B sperm, respectively, compared to wild-type mice, alongside a notable increase of 64.63% in grade D sperm. This had a significant detrimental impact on mouse fertility, as evidenced by transmission electron microscopy (TEM) revealing severe damage to the sperm plasma membrane. The secretion of LDHA by SCs plays a crucial role in preserving the quantity and quality of round and elongated sperm cells, safeguarding sperm motility, and maintaining the integrity of the plasma membrane, thereby influencing spermatogenesis. Within SCs, Glucose transporter 3 (GLUT3) functions as a pivotal transporter responsible for directing glucose towards lactic acid production. In the study by Zhang et al. (53), it was observed that GLUT3 expression markedly declined following the in vitro overexpression of mmu-miR-320-3p. This reduction led to pronounced apoptosis of sperm cells and spermatocytes, culminating in impaired fertility potential in mice. These findings suggest that the disruption of the mmu-miR-320-3p/GLUT3 pathway, critical for lactate production in SCs, directly caused a decrease in lactate secretion by SCs, ultimately resulting in disturbances in spermatogenesis. Nevertheless, the secretion patterns of lactic acid and estradiol (E2) in primates exhibit variances compared to those in rats. SCs in untreated monkeys have the capacity to independently generate substantial quantities of lactic acid; however, there is no augmentation in lactic acid secretion observed when SC is exposed to high doses of recombinant human follicle-stimulating hormone (rmFSH) or testosterone (T)/rmFSH. Likewise, in a culture setting, T can undergo conversion to E2 without the requirement for rmFSH intervention (54). The primate secretion of spermatogenesis-related nutrient substrates, including lactic acid and E2, in an FSH-independent manner, broadens the scope of therapeutic considerations for individuals with idiopathic infertility in clinical settings. Secretions that Sertoli cells during the process of spermatogenesis can be seen in **Figure 1**.

## 5 Sertoli cell secretions affect spermatogenesis by acting on themselves

**Figure 2:**
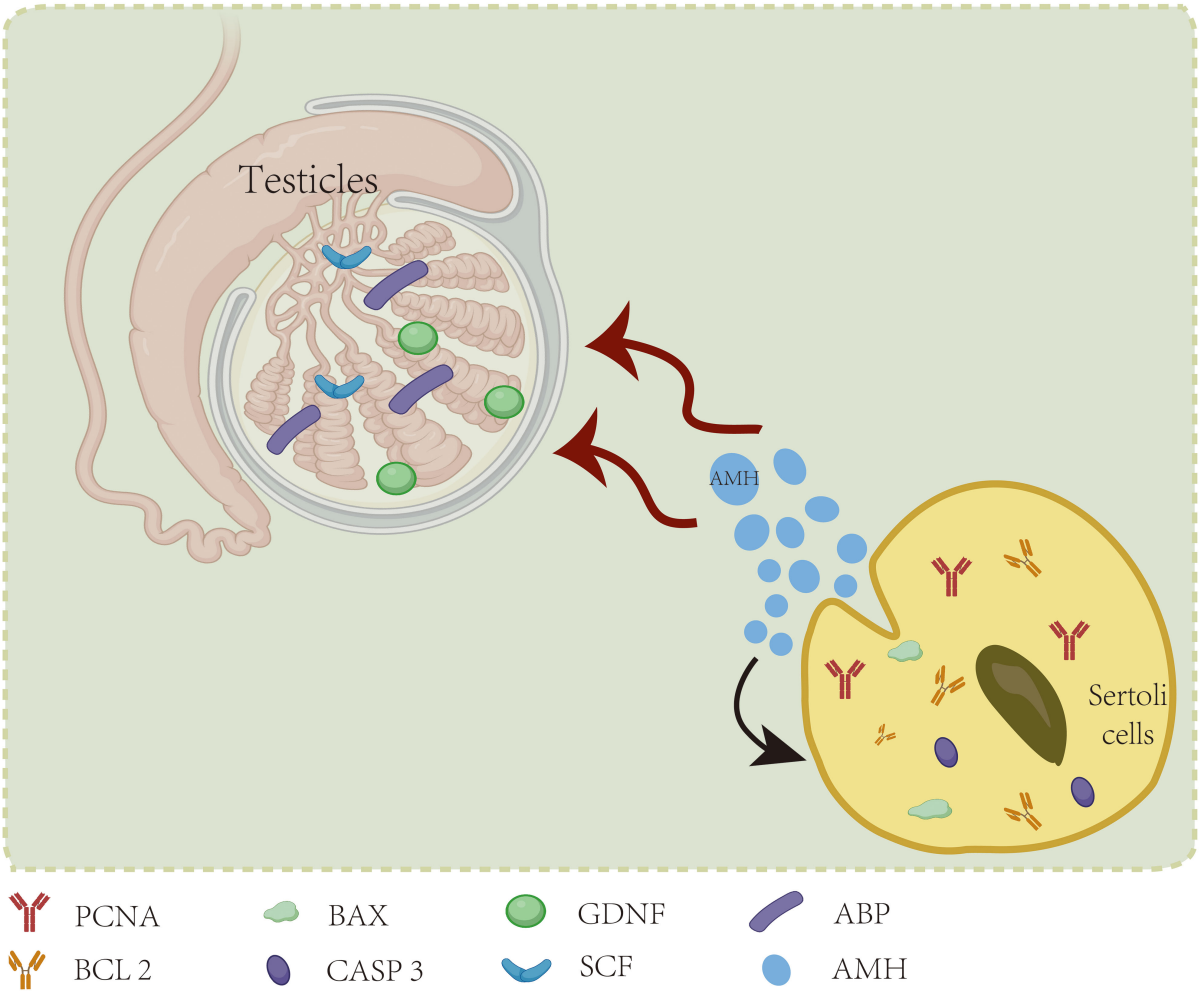
SC secretions by acting on themselves.

Research has revealed that anti-Mullerian hormone (AMH) also directly influences the fate of SCs themselves. In a pioneering study by Qin et al. (55), the impact of AMH secreted by yak SCs on SC development and its role in yak spermatogenesis was investigated. Silencing AMH expression in yak SCs via RNA interference led to reduced levels of PCNA and BCL2 genes associated with SC proliferation, along with elevated expression of BAX and CASP3 genes linked to apoptosis. Furthermore, the expression of the specific androgen receptor (AR) gene in mature SCs exhibited a notable decrease of 21.8%, underscoring the essential role of AMH in the development and function of yak SCs. Additionally, the mRNA levels of key genes implicated in spermatogenesis, including GDNF, SCF, and ABP, were downregulated, indicating that disrupted AMH secretion in SCs adversely impacted normal spermatogenic processes. In a study by Rehman et al. (56), a 48-hour in vitro intervention was carried out using recombinant human anti-Mullerian hormone (rh-AMH) at varying concentrations of 0, 10 ng/ml, 50 ng/ml, 100 ng/ml, and 800 ng/ml with mouse SCs. The expression levels of caspase-3 and the pro-apoptotic protein Bax demonstrated a positive correlation with the concentration of rh-AMH, exhibiting a concentration-dependent decrease in anti-apoptotic protein Bcl2. Moreover, the mRNA expression level of Scf showed a six-fold increase compared to the control group upon treatment with a low concentration (10 ng/ml) of recombinant human anti-Mullerian hormone (rh-AMH), with the most pronounced SCF mRNA elevation observed following treatment with a moderate concentration (50 ng/ml) of rh-AMH. In addition to the intrinsic autocrine and paracrine regulation of AMH by the cells themselves, AMH can reciprocally influence spermatogenesis and testicular development. Furthermore, significant variations in AMH levels were observed in cryptorchidism patients aged 2 years. In a controlled clinical trial involving 54 participants (57), notable reductions in AMH and statin B levels were observed in boys with cryptorchidism. Specifically, among the cohort, the expression levels of AMH and statin B in 10 children with bilateral cryptorchidism decreased to more than twice that of the normal control group. A strong positive correlation was evident between AMH and statin B levels in this context. After age adjustment and considering the compensatory effect of the healthy testis side, the AMH secretion levels in the remaining 17 children with unilateral cryptorchidism did not show significant differences compared to the control group. This implies that the secretion levels of AMH and statin B could serve as valuable indicators to guide the clinical management of children with cryptorchidism. In a clinical investigation conducted by Rune Holt et al. (58) involving 307 infertile male patients, it was demonstrated that serum AMH levels may serve as an indicator of impaired supporting cell and spermatogenesis functions. However, in fertile male populations, serum AMH levels were not found to be directly associated with spermatogenesis. The impact of Sertoli cell secretions on themselves can be seen in **Figure 2**.

## 6 Others

Exosomes released by SCs have the capacity to traverse the blood-testis barrier and reach stromal cells. In a study by Ma et al. (59), it was observed through an in vitro co-culture experiment of SCs and Leydig cells (LC) that SCs conveyed CC-chemokine ligand 20 (Ccl20) mRNA to LC via exosome secretion. This process stimulated the phosphorylation of protein kinase B (AKT) in interstitial cells by upregulating protein and mRNA expression levels. Consequently, this process enhances the viability of interstitial cells and boosts testosterone secretion. In a study by Huang et al. (60), exposure of SC-derived exosomes (SC-Exo) to 5mg/kg/bw perfluorooctane sulfonic acid (PFOS) led to a significant elevation in the expression level of miR-9-3p within SC-Exo. This increase resulted in the downregulation of its downstream target, steroidogenic acute regulatory protein (StAR). Inhibition of testosterone synthesis in testicular stromal cells can be achieved through the transfer of miR-9-3p from SCs to these cells via exosomes. Extensive experimental evidence has demonstrated that exosomes play a crucial role in mediating the transmission of miR-9-3p from SCs to testicular stromal cells, consequently impacting spermatogenesis by suppressing testosterone synthesis. Recent investigations have revealed that miR-145-5p, released by immature Sertoli cells (ISC) and mature Sertoli cells (ASC), exerts a bidirectional regulatory influence on steroidogenic genes and testosterone production in testicular stromal cells (61). Highly expressed in ISC, miR-145-5p facilitates the targeting of ISC-EXO on steroidogenic factor-1 (Sf-1), resulting in lipid droplet accumulation in stromal cells through the downregulation of steroidogenic genes. This process effectively hinders testosterone synthesis. Conversely, miR-145-5p exhibits low expression in ASC, yet it exerts a negative feedback mechanism on LC by enhancing the expression of steroidogenic genes, thereby stimulating testosterone production.

Clusterin is typically present on the surface of aberrant sperm and is considered a hallmark of pathological sperm (62). Following secretion by SCs, clusters of clusterin adhere to the cell membrane of elongated and mature sperm cells through the fluid within the spermatogenic epithelium. In the study conducted by K. Matsushita et al. (63), rat testes were subjected to immersion in warm water at 43°C for 15 minutes. It was observed that after 4 days, the testicular volume was notably reduced compared to the control group, and by the 7th day, the number of germline cells had gradually decreased by 80%. Concurrently, there was a time-dependent increase in the expression of Clusterin mRNA. Upon silencing the upregulation of clusterprotein using siRNA-Clu, SCs exhibited a higher percentage of apoptosis compared to the control group. Numerous experiments have substantiated that Clusterin, acting as a potential protective factor for testicular cells, suppresses germ cell apoptosis and safeguards normal spermatogenesis under conditions of testicular heat stress by inducing upregulation of its expression. The concentration of clusterin in semen exhibits a positive correlation with its concentration in the testes and a negative correlation with the severity of spermatogenic disorders. It can be inferred that the expression level of clusterin in semen may serve as a reflection of the spermatogenic function in males (64).

## 7 Summary

Male infertility research has traditionally centered on fertility issues stemming from abnormal sperm. The smooth progression of spermatogenesis significantly impacts the generation of viable sperm. While supportive cells have garnered attention for their roles as "nutrients" and "scaffolders", their secretory functions and the substances they release are equally crucial for male fertility. This review compiles the diverse physiological and pathological impacts of SC secretions across the entirety of spermatogenesis, highlighting the robust association between aberrant spermatogenesis and Sertoli cell secretions. Despite the incomplete understanding of the function and mechanisms of cytosecretory support substances due to technical constraints and methodological limitations, the current research outcomes present novel avenues for addressing certain refractory and targeted infertility issues. Further investigations in this domain have the potential to unveil new targets and therapeutic strategies for androgenic disorders arising from abnormal spermatogenesis.

In conclusion, the deepening comprehension of SC secretions emphasizes the significance of their autocrine and paracrine roles. Future investigations should focus on more targeted studies to unveil the intricacies of SC secretory functions and advance developments in the field of male reproductive health.
